# Smoking related lung cancer mortality by education and sex in Norway

**DOI:** 10.1186/s12885-019-6330-9

**Published:** 2019-11-21

**Authors:** Merethe S. Hansen, Idlir Licaj, Tonje Braaten, Arnulf Langhammer, Loic Le Marchand, Inger Torhild Gram

**Affiliations:** 1 Department of Community Medicine, Faculty of Health Sciences, The UiT Arctic University of Norway, Tromsø, Norway; 2 Clinical Research Department, Centre François Baclesse, Caen, France; 3 Department of Public Health and Nursing, NTNU, Norwegian University of Science and Technology, Trondheim, Norway; 4 Cancer Epidemiology Program, University of Hawai`i Cancer Center-University of Hawai`i at Manoa, Honolulu, HI USA

**Keywords:** Cohort study, Lung cancer mortality, Smoking, Education, Sex differences

## Background

Lung cancer is one of the most common forms of cancer and the leading cause of cancer death worldwide, with tobacco smoking as the main cause [1]. In Norway, as in other western countries, smoking was more prevalent among men and in the highest social classes six to seven decades ago [2]. The proportion of male smokers increased until the 1960s, when it was around 65%. Among women, the peak (35%) occurred in the late 70’s [2]. From 1930 until the turn of the century, men have consumed more than 70% of the cigarettes smoked in Norway [3]. The decline in smoking prevalence occurred first and proceeded fastest among those with long education [4]. In Norway, lung cancer mortality for men has been declining since 2011, whereas as of 2013 it is still increasing for women [5]. Due to the lag period between start of smoking and lung cancer death, current mortality rates reflect smoking trends two to three decades earlier [6].

Neither the most recent World Cancer Report [1] nor the United States Surgeon General Report [7] discuss a possible sex difference in the risk of smoking associated lung cancer mortality. In 2001, Tverdal reported that among Norwegians under 50 years of age, lung cancer mortality was higher in women than in men [8]. Later Jha et al. reported from a US cohort, that among current compared with never smokers, women had a higher lung cancer mortality compared with men [9]. Since men and women have entered the stages of the smoking epidemic at different calendar times [10], a possible sex difference for smoking and lung cancer mortality may just have started to emerge. Education, an indicator of socioeconomic status is inversely associated with cancer mortality [11, 12].

Studies from Europe have reported an increased risk of lung cancer in participants of low socioeconomic status despite accounting for smoking habits [13, 14]. To our knowledge, no other prospective cohort studies have examined lung cancer mortality by sex and education.

The objectives of the study were to explore a potential heterogeneity in smoking associated lung cancer mortality by sex and education.

## Methods

### Study population

The study population has been previously described [15] and comprises three national Norwegian health studies conducted between 1974 and 2003 by the Norwegian National Health Screening Service. Selection of participants was based on year of birth and residence (municipality or county). The response rate in the three studies varied from 56 to 88% [16]. Briefly, the three surveys used a similar protocol and study design, but there were some modifications made during different time periods, mainly due to questionnaires regarding smoking, physical activity and other lifestyle factors. Altogether 595,675 participants remained in the analytical cohort after exclusion of 40,091 participants due to emigration or death before the start of follow-up, missing information on vital status, measures of smoking exposure, education, or missing of any of the covariates included in the analyses.

The present study was approved by the Regional Committee for Medical Research Ethics South-East, Norway, and the National Data Inspectorate.

### Exposure information

The questionnaires elicited information on current and former daily smoking, smoking duration in years (continuous), and average number (continuous) of cigarettes smoked per day.

Among the 373,283 ever smokers in our sample, the proportion of missing values was 5% (*n* = 18,886) for smoking duration, number of cigarettes per day, and pack-years (i.e., number of cigarettes smoked per day, divided by 20, multiplied by the smoking duration in years).

We categorized current smokers according to smoking duration in years (1–19, 20–29, ≥30), number of cigarettes smoked per day (1–10, 11–20, > 20), and pack-years (1–9, 10–19, ≥20).

We classified participants by level of education into three categories: < 10, 10–12, and ≥ 13 years by using the most recent information regarding duration of education obtained from Statistics Norway. We classified for physical activity in three: [sedentary (reading, watching television, and sedentary activity), moderate (walking, bicycling, and/or similar activities ≥4 h per week), and heavy (light sports or heavy gardening ≥4 h per week, heavy exercise, or daily competitive sports)] categories. We calculated BMI as weight in kg divided by height in m^2^ and classified in three and classified in three (< 18.5 kg/m^2^, 18.5–24.9 kg/m^2^, ≥25.0 kg/m2) categories. All variables were obtained at study enrollment. As questions on alcohol consumption were only included from 1994 onwards, information on alcohol consumption was missing in 73% of the participants in the analytical cohort.

### Follow-up and endpoints

The data were linked to the Cancer Registry of Norway, the Norwegian Cause of Death Registry, and the Central Population Register by the national, unique 11-digit personal identification number. Lung cancer mortality was classified according to the eight, ninth and tenth revisions of The International Classification of Diseases (ICD-8, ICD-9, ICD-10). Follow-up ended at the time of death from primary lung cancer, death from any other causes, emigration, or the end of follow-up (December 31, 2013), whichever occurred first.

All deaths connected to primary incident carcinomas of the trachea, bronchus, and lung (ICD-8 code 162 or corresponding codes from ICD-9 and ICD-10) were included as endpoint, i.e. death from lung cancer.

### Statistical analysis

We calculated the age-standardized (European Standard Population) overall lung cancer mortality rate by smoking status, and categories of education [17].

We used Cox proportional hazards model with attained age between cohort entry and exit as the underlying time scale to estimate the multivariable-adjusted hazard ratios (HRs) with 95% confidence intervals (CI), for the associations between different measures of smoking exposure and lung cancer mortality. We used stratified Cox models by cohort study and birth cohort (≤ 1950 and > 1950) to overcome any probable heterogeneity for these variables. A priori we considered alcohol, physical activity, BMI and education as possible confounders. We tested for interaction between smoking status and sex, and between smoking and education, and decided to stratify by sex and by education. We decided to adjust on BMI and physical activity, but did not include alcohol as a covariate because of a lot of missing data. We estimated dose-response associations among current smokers for the following variables measured continuoulsy: smoking duration in 10 years, number of 10 cigarettes smoked per day, number of 10-pack-years, and lung cancer mortality overall. Never smokers were not inluded in these analyses.

Subsequently, we tested for linear trend for smoking exposure (smoking duration, cigarettes smoked per day and pack-years) among current smokers based on the median value in each category, using the lowest category of each measure of smoking exposure as reference.

We used the Wald test to assess heterogeneity by sex and by education for the associations between different measures of smoking exposure and lung cancer mortality. We tested and found that the criteria for the proportional hazard assumption were met using Schoenfeld residuals (data not shown).

Subsequently, we performed the same analyses after excluding individuals who died from lung cancer within < 2 years of follow-up, and we also performed the same analyses after excluding participants with prevalent cancer.

We conducted all analyses using STATA version 14.0 (Stata Corp.). We considered two-sided *p*-values of < 0.05 as statistically significant.

## Results

During the nearly 12 million (48% men) person-years of observation and an average of 19 years of follow-up, we identified 5702 (58% men) lung cancer deaths. Altogether 39% were current, 24% former and 37% never smokers at enrollment. The majority (55%) of participants had from 10 to 12 years of education, 23% had less than 10 years, and 22% had 13 years or more. The overall crude LC mortality rate was 6,1 per 100,000 among never, 23,9 per 100.000 among former and 99,2 per 100.000 among current smokers. The corresponding rates for those at the lowest, middle and highest level of education was 87,6 per 100,000, 38,7 per 100,000 and 20,4 per 100,000, respectively. There was an interaction between smoking and sex (*P* < 0.0001), and a borderline interaction between smoking and education (*P* = 0.06).

Table 1 shows that compared with women, men were more likely to be ever (current or former) smokers, and to have smoked more pack-years for all three levels of education. The proportion of never smokers were 41% in women and 33% in men. More men (23%) than women (20%) were in the highest level of education. Women with the longest education had the highest (57%) proportion of never smokers. Among both men and women the number of lung cancer deaths was highest in the less educated (Table 1).

**Table 1:** Characteristics of the study population by education, the Norwegian Health Screening Surveys, 1974–2003, (*N* = 595,675)

Characteristics	Education in years							
	< 10		10–12		≥13		All	
	Men	Women	Men	Women	Men	Women	Men	Women
Subjects (%)	64,024 (22)	76,455 (25)	155,905 (55)	169,949 (55)	66,332 (23)	63,010 (20)	286,261 (48)	309,414 (52)
Lung cancer cases^a^, n (%)	1646 (44)	1338 (47)	1759 (46)	1297 (45)	385 (10)	238 (8)	3790	2873
Lung cancer deaths, n (%)	1517 (46)	1138 (48)	1473 (44)	1056 (44)	333 (10)	185 (8)	3323	2379
Person-years of follow up	1,365,688	1,666,446	3,106,850	3,429,805	1,314,443	1,224,279	5,786,981	6,320,530
Body mass index (mean, SD)	26 (3)	25 (4)	26 (3)	25 (4)	25 (3)	24 (4)	26 (3)	25 (4)
Heavy physical activity^b^ (%)	28	14	36	22	41	28	35	20
Never smokers (%)	20	33	32	38	50	57	33	41
Former smokers (%)	27	18	27	22	26	23	27	21
Current smokers (%)	53	49	41	40	24	20	40	38
Duration of smoking^c^, years, median (interquartile range)	22 (15–26)	20 (15–25)	20 (13–25)	19 (10–23)	18 (10–22)	15 (8–20)	20 (13–25)	20 (10–23)
Cigarettes smoked per day^c^, median (interquartile range)	15 (10–20)	10 (8–15)	15 (10–20)	10 (7–15)	12 (10–20)	10 (5–15)	15 (10–20)	10 (7–15)
Pack-years^c^, median (interquartile range)	14 (8–21)	10 (5–16)	13 (7–20)	9 (4–15)	10 (5–18)	6 (3–12)	13 (7–20)	9 (4–15)
Age at enrollment, median (interquartile range)	42 (40–45)	42 (41–45)	41 (40–42)	41 (40–42)	42 (41–43)	41 (40–42)	41 (40–42)	41 (40–43)
Age at lung cancer death, never smokers, median (interquartile range)	75 (62–80)	77 (68–84)	62 (52–73)	64 (57–76)	57 (55–69)	61 (55–66)	63 (54–76)	66 (59–80)
Age at lung cancer death, current smokers, median (interquartile range)	66 (60–74)	63 (57–71)	63 (57–70)	60 (55–66)	64 (58–70)	61 (56–68)	64 (58–72)	62 (56–69)

^a^At enrollment

^b^Heavy physical activity: light sports or heavy gardening ≥4 h/week, heavy exercise or daily competitive sports

^c^Duration of smoking, cigarettes smoked per day and pack-years in ever smokers

Additional file 1: Table S1 shows that the mean age at enrollment was 40, 43 and 48 in the Norwegian Counties Study, the 40 years Study and Cohort of Norway (CONOR) respectively. The Norwegian Counties Study was characterized by lower level of education and higher proportion of current smokers than the 40 years study and CONOR (Additional file 1: Table S1).

Table 2 shows that compared with sex-specific never smokers, current smokers had a lung cancer mortality hazard ratio of 20.05 (95% CI 16.25–24.74) for men, and 13.97 (95% CI 11.98–16.29) for women (*P*_heterogeneity_ = 0.01). For each 10-years increase in smoking duration women had a 65% higher hazard ratio [HR: 1.65 (95% CI 1.54–1.78)] and men a 36% higher HR [HR: 1.36 (95% CI 1.28–1.44)] (*P*_heterogeneity_ < 0.01). For women compared with men, current smokers had a greater increase in lung cancer mortality per unit of number of cigarettes per day and number of pack-years (Both *P*_heterogeneity_ < 0.01) (Table 2).

**Table 2:** Hazard ratios^a^ for lung cancer mortality according to smoking status and continuous measures of exposure

Smoking status	Cases	Men HR 95%CI	Cases	Women HR 95%CI	Heterogeneity test for men versus women *P*-values
Never	91	1.00 (ref)	188	1.00 (ref)	
Former	459	4.08 (3.25–5.11)	208	2.71 (2.22–3.30)	0.01
Current	2773	20.05 (16.25–24.74)	1983	13.97 (11.98–16.29)	0.01
Duration of smoking, 10-years					
b	2761	1.36 (1.28–1.44)	1969	1.65 (1.54–1.78)	< 0.01
Cigarettes smoked per day, 10 per day					
^b^	2676	1.48 (1.42–1.54)	1974	1.76 (1.66–1.86)	< 0.01
Pack-years (10 years)					
^b^	2269	1.39 (1.35–1.44)	1965	1.61 (1.54–1.69)	< 0.01

^a^ Multivariable Hazard ratios (95% CI) adjusted for body mass index, physical activity level, all at enrollment, and level of education

^b^Per 10-year increase in smoking duration, per 10-cigarettes increase number of cigarettes smoked per day, per 10 increase

in pack-years, for current smokers

Additional file 2: Table S2 shows the multivariable HR for lung cancer mortality according to categorical measures of smoking exposure for current smokers by sex compared with sex specific never smokers. The estimates did not vary much by sex, except that men who had smoked < 20 years, had a higher HR [HR: 11.78 (95% CI 9.26–14.98)] compared with women [HR: 7.29 (95% CI: 6.05–8.78)] (*P*_heterogeneity_ < 0.01). For those who had smoked less than 10 pack-years, men had a higher HR compared with women (*P*_heterogenety_ = 0.02) (Additional file 2: Table S2).

Table 3 shows that among never smokers, women with the lowest level of education had the highest age-adjusted lung cancer mortality rate which was (16.7 per 100,000 person-years). The highest rate was among the less educated current smokers for both men (319.0 per 100.000 person-years) and women (183.0 per 100,000 person-years). For all three levels of education, males had a higher lung cancer mortality rate compared with females for both former and current smokers (Table 3).

**Table 3:** Age adjusted^a^ lung cancer mortality rates per 100,000 person-years by education and smoking status

Smoking status	Men			Women		
	Education in years^b^					
	< 10	10–12	≥13	< 10	10–12	≥13
Never	8,6	9,7	6,8	16,7	8,9	8,3
Former	83,8	56,7	51,0	47,2	26,5	24,6
Current	319,0	208,8	194,2	183,0	133,1	102,6

^a^Age adjusted according to the European Standard Population

^b^Education: < 10 years, 10–12 years, ≥13 years

Table 4 shows that for male current smokers the HR did not vary for the different categories of smoking exposure when we compared those with the lowest and highest level of education (all *P*_heterogeneity_ > 0.05). For female current smokers there was a significant difference between those with < 10 years [HR: 15.85 (95% CI 12.32–20.38)] compared with those with ≥13 years of education [HR: 9.41 (95% CI 6.49–13.68)] (*P*_heterogeneity_ < 0.01). For female current smokers the HR in the lowest category for the three smoking exposures (duration of smoking, cigarettes smoked per day and pack-years) were significantly higher when we compared those with the lowest and highest level of education (all *P*_heterogeneity_ < 0.02) (Table 4).

**Table 4:** Hazard ratios for lung cancer mortality in current smokers, by smoking exposure and education

Men^a^ HRs 95% CI							
	Education in years						
Smoking status	Cases	< 10 years HR^a^ 95% CI	Cases	10–12 years HR^a^ 95% CI	Cases	≥13 years HR^a^ 95% CI	Heterogeneity test^b^ *P*-values
Never smokers^c^	18	1.00 (ref)	54	1.00 (ref)	19	1.00 (ref)	
Current smokers	1303	28.96 (18.17–46.14)	1216	16.01 (12.19–21.05)	254	22.50 (14.09–35.92)	0.45
Duration of smoking (years)							
1–19	120	18.27 (11.08–30.12)	130	9.13 (6.61–12.61)	27	9.95 (5.49–18.03)	0.13
20–29	717	27.93 (17.43–44.74)	758	15.61 (11.80–20.66)	151	23.56 (14.51–38.25)	0.62
> 30	465	32.72 (20.35–52.62)	318	19.92 (14.65–27.09)	75	35.32 (19.59–63.69)	0.84
P for trend^d^		< 0.01		< 0.01		< 0.01	
Cigarettes smoked per day							
1–10	400	20.71 (12.91–33.23)	262	8.98 (7.00–12.05)	53	12.26 (7.25–20.74)	0.15
11–20	684	34.57 (21.62–55.28)	710	19.00 (14.38–25.10)	142	27.96 (17.27–45.29)	0.54
> 21	165	54.57 (33.46–89.00)	211	33.76 (24.96–45.65)	49	50.24 (29.37–85.93)	0.82
P for trend^d^		< 0.01		< 0.01		< 0.01	
Pack-years							
1–9	141	15.84 (9.68–25.92)	101	6.49 (4.65–9.05)	19	7.34 (3.88–13.91)	0.06
10–19	497	25.45 (15.88–40.78)	443	13.55 (10.20–18.00)	87	21.18 (12.85–34.89)	0.60
≥ 20	611	40.43 (25.28–64.65)	632	23.84 (18.04–31.54)	138	37.02 (22.78–60.17)	0.80
P for trend^d^		< 0.01		< 0.01		< 0.01	
Women^a^ HRs 95% CI							
Smoking status	Cases	HR^a^ 95% CI	Cases	HR^a^ 95% CI	Cases	HR^a^ 95% CI	
Never smokers	70	1.00 (ref)	81	1.00 (ref)	37	1.00 (ref)	
Current smokers	980	15.85 (12.32–20.38)	887	14.22 (11.28–17.92)	116	9.41 (6.49–13.68)	< 0.01
Duration of smoking (years)							
1–19	155	8.37 (6.21–11.29)	158	7.62 (5.78–10.06)	20	3.83 (2.20–6.65)	0.01
20–29	624	16.11 (12.32–21.08)	603	16.19 (12.70–20.63)	73	11.01 (7.35–16.48)	0.12
> 30	194	23.05 (17.16–30.97)	120	21.85 (15.79–30.25)	22	27.18 (13.30–55.52)	0.68
P for trend^d^		< 0.01		< 0.01		< 0.01	
Cigarettes smoked per day							
1–10	458	12.81 (9.87–16.62)	326	9.43 (7.37–12.07)	36	5.15 (3.26–8.20)	< 0.01
11–20	465	22.88 (17.50–29.92)	500	21.35 (16.75–27.22)	78	14.47 (9.66–21.69)	0.06
> 21	54	41.62 (28.75–60.25)	57	39.87 (28.22–56.34)	8	19.70 (9.04–42.97)	0.09
P for trend^d^		< 0.01		< 0.01		< 0.01	
Pack-years							
1–9	197	8.31 (6.27–11.02)	148	6.08 (4.61–8.01)	18	3.31 (1.88–5.84)	< 0.01
10–19	481	18.27 (14.05–23.77)	457	16.95 (13.31–21.60)	57	12.00 (7.89–18.25)	0.10
≥ 20	294	29.66 (22.60–38.93)	274	27.92 (21.66–35.98)	39	18.49 (11.68–29.26)	0.08
P for trend^d^		< 0.01		< 0.01		< 0.01	

^a^ Multivariable Hazard ratios (95% CI) adjusted for body mass index and physical activity, both at enrollment

^b^Heterogeneity test for those with < 10 years of education compared with ≥13 years education

^c^Never smokers

^d^Trend test without never smokers

The results did not change substantially when we excluded individuals who died from lung cancer within < 2 years of follow-up. The results stayed the same when we excluded those with prevalent cancer at enrollment (data not shown).

## Discussion

In this large Norwegian cohort study, we found that more men were current or former smokers, more were heavy smokers and more smokers had died from lung cancer, regardless of level of education, compared with women. For both men and women, those with the lowest compared with the highest level of education, were more likely to die from lung cancer regardless of smoking status. However, when we analyzed the three smoking exposure measures for current smokers as continuous variables, female smokers seem to be more likely to die from lung cancer, for increments of 10 years of smoking, 10 cigarettes/day and 10 pack-years compared with male smokers.

Our results are in line with those of other prospective cohort studies [18–21] and a meta-analysis of three prospective cohort studies [22], which have found that compared with females, males are heavier smokers and die more from lung cancer. In the present study, we observed a difference in lung cancer mortality between male and female smokers, while several other cohorts did not [18–23]. These studies did not use continuous measures for smoking exposure as we did, but rather broad categories for number of cigarettes smoked per day. Thus men may be in the upper and women in the lower part of a specific category, but still be classified as being similarly exposed. The US cohort, with 17,670 cases, found a virtually identical lung cancer mortality rate for male and female current smoker in the most recent time periods, while for the earliest cohorts they observed a higher risk for men, reflecting the differences in smoking prevalence by sex [22], and the stages of the smoking epidemic by sex described earlier [10]. Since lung cancer mortality rates for Norwegian women have not peaked yet, they may become higher than that for the US women, which already in 2001 was warned by Tverdal [8]. Jha et al. [24], have pointed out that the full effects of smoking can take 50 years to measure in individuals, and up to 100 years to measure in populations. The results from the present study and from that of Tverdal [8], both showing sex differences in Norway, may be early indicators of this long-term development of sex differences in smoking related lung cancer mortality. Other indicators that the sex difference in smoking related lung cancer mortality in the long-term effect of smoking are our [15], and those of the US cohort [9]. An alternative explanation for the higher lung cancer mortality in smoking females compared with men in our study may be competing risk of death. Since men smoke more than women, they have increased risk for dying of other smoking-related diseases before they get lung cancer.

In Norway, there is a marked social gradient for active as well as passive smoking. The lower the education, the more smoking [4]. As expected, the age standardized rates of lung cancer mortality were highest in the less educated male and female current smokers. For both men and women, our results indicate that the less educated had a higher lung cancer mortality compared with the highly educated. The difference by level of education for both men and women should be interpreted with caution, as this could be due to residual confounding by smoking as there was a large proportion of heavy smoking men and women, in the less educated. Another explanation for smoking related difference in lung cancer mortality by both sex and education could be related to measures of socioeconomic status like passive smoking from spouses, radon, occupational exposure and air pollution. Similarly, studies from the EPIC (European Prospective Investigation into Cancer and Nutrition) and Sweden, respectively, observed a higher risk of lung cancer in the lower social class despite accounting for smoking habits [13, 14].

Among never smokers, we observed that both men and women in the lowest level of education died more from lung cancer compared with their counterparts in the highest level of education. A possible explanation may be residual confounding by smoking as well as exposure to occupational and passive smoking exposure.

Our study has several major strengths. It is based on a large, prospective Norwegian cohort, comprising a high proportion of male and female ever smokers, with long, virtually complete follow-up. The questions on smoking duration and number of cigarettes per day allowed respondents to give open-ended answers which allowed us to utilize continuous measures of smoking exposure. Moreover, we have more than 5500 lung cancer deaths, yielding higher precision of the estimates and power to discover a true difference.

One limitation is that we only have information on smoking and other potential confounders at study enrollment. Another limitation is that we lack information on passive and occupational smoking.

Around 10% of the Norwegian population reported to be occasional smokers in our follow-up period [25]. Some of them may have been included as never smokers, which most likely will have attenuated the observed associations between smoking and lung cancer death. We do not believe that these limitations would distort the smoking related sex difference in lung cancer mortality revealed in our study.

## Conclusion

Our findings, in this large cohort study, suggest that women have increased risk of dying from lung cancer compared with men, given the same smoking history. In addition, low education confers an increased risk of dying from lung cancer, which could be due to residual confounding by active and passive smoking.

## Supplementary information

**Additional file 1: Table S1.** Selected characteristics of the study population at enrollment, stratified by cohort, (*N* = 595,675).

**Additional file 2: Table S2.** Hazard ratios^a^ (95% CIs) for lung cancer mortality according to categorical measures, for current smokers.

## Data Availability

The dataset used during the current study are available from the corresponding author on reasonable request.

## References

[1] Steward BW, Wild CP. World Cancer Report 2014.: International Agency for Research on Cancer World Cancer Report; 2014. 39432694

[2] Lund KE, Lund M, Bryhni A (2009). Tobacco consumption among men and women 1927-2007. Tidsskr Nor Laegeforen.

[3] Lund I, Lund KE (2014). Lifetime smoking habits among Norwegian men and women born between 1890 and 1994: a cohort analysis using cross-sectional data. BMJ Open.

[4] Næss Ø, Rognerud M, Strand BH. Sosial ulikhet i helse. En faktarapport FHI. Oslo Folkehelseinstituttet. 2007.

[5] Cancer Registry of Norway. Cancer in Norway 2013. Oslo, Norway: Cancer Registry of Norway 2013 [updated March, 2015; cited 2016 03.10]. Available from: https://www.ssb.no/en/helse/statistikker/royk/aar/2014-02-05.

[6] Islami F, Torre LA, Jemal A (2015). Global trends of lung cancer mortality and smoking prevalence. Translational lung cancer research.

[7] National Center for Chronic Disease P, Health Promotion Office on S, Health. Reports of the Surgeon General. The Health Consequences of Smoking-50 Years of Progress: A Report of the Surgeon General. Atlanta (GA): Centers for Disease Control and Prevention (US); 2014.

[8] Tverdal A (2001). Lung cancer mortality--now higher in women than in men under 50 years. Tidsskr Nor Laegeforen.

[9] Jha P, Ramasundarahettige C, Landsman V, Rostron B, Thun M, Anderson RN (2013). 21st-century hazards of smoking and benefits of cessation in the United States. N Engl J Med.

[10] Thun M, Peto R, Boreham J, Lopez AD (2012). Stages of the cigarette epidemic on entering its second century. Tob Control.

[11] Hart CL, Hole DJ, Gillis CR, Smith GD, Watt GC, Hawthorne VM (2001). Social class differences in lung cancer mortality: risk factor explanations using two Scottish cohort studies. Int J Epidemiol.

[12] Braaten T, Weiderpass E, Lund E (2009). Socioeconomic differences in cancer survival: the Norwegian women and Cancer study. BMC Public Health.

[13] Ekberg-Aronsson M, Nilsson PM, Nilsson JA, Pehrsson K, Lofdahl CG (2006). Socio-economic status and lung cancer risk including histologic subtyping--a longitudinal study. Lung Cancer.

[14] Menvielle G, Boshuizen H, Kunst AE, Dalton SO, Vineis P, Bergmann MM (2009). The role of smoking and diet in explaining educational inequalities in lung cancer incidence. J Natl Cancer Inst.

[15] Hansen MS, Licaj I, Braaten T, Langhammer A, Le Marchand L, Gram IT. Sex differences in risk of smoking- associated lung Cancer: results from a cohort of 600,000 Norwegians. Am J Epidemiol. 2017. 10.1093/aje/kwx339 29087432

[16] Stocks T, Borena W, Strohmaier S, Bjorge T, Manjer J, Engeland A (2010). Cohort profile: the metabolic syndrome and cancer project (me-can). Int J Epidemiol.

[17] Geography PDT (2018). Standard populations. In: Scotland NS, editor.

[18] Vollset SE, Tverdal A, Gjessing HK (2006). Smoking and deaths between 40 and 70 years of age in women and men. Ann Intern Med.

[19] Nilsson S, Carstensen JM, Pershagen G (2001). Mortality among male and female smokers in Sweden: a 33 year follow up. J Epidemiol Community Health.

[20] Jamrozik K, McLaughlin D, McCaul K, Almeida OP, Wong KY, Vagenas D (2011). Women who smoke like men die like men who smoke: findings from two Australian cohort studies. Tob Control.

[21] Prescott E, Osler M, Andersen PK, Hein HO, Borch-Johnsen K, Lange P (1998). Mortality in women and men in relation to smoking. Int J Epidemiol.

[22] Thun MJ, Carter BD, Feskanich D, Freedman ND, Prentice R, Lopez AD (2013). 50-year trends in smoking-related mortality in the United States. N Engl J Med.

[23] Marang-van de Mheen PJ, Smith GD, Hart CL, Hole DJ (2001). Are women more sensitive to smoking than men? Findings from the Renfrew and Paisley study. Int J Epidemiol.

[24] Jha P (2009). Avoidable global cancer deaths and total deaths from smoking. Nat Rev Cancer.

[25] Norwegian Institue of Public Health. Public Health Report 2014 Oslo2014 [updated 12.04.2016; cited 2016 03.10]. Available from: https://www.fhi.no/en/.

