# Supplementary material for: Smoking related lung cancer mortality by education and sex in Norway
Source: BMC Cancer. 2019 Nov 21;19:1132. doi: 10.1186/s12885-019-6330-9 (PMC6873553; doi:10.1186/s12885-019-6330-9)
Supplement: Supplementary file 1 — Additional file 1: Table S1. Selected characteristics of the study population at enrollment, stratified by cohort, (N = 595,675). [file 12885_2019_6330_MOESM1_ESM.docx]

Table S1. Selected characteristics of the study population at enrollment, stratified by cohort, (N=595,675)

|  | Norwegian Counties Stud  (1974-1987)^a^ | | 40 years Cohort  (1985-1999)^a^ | | Cohort of Norway (CONOR)  (1994-2003)^a^ | |
| --- | --- | --- | --- | --- | --- | --- |
|  | Men | women | Men | Women | Men | Women |
| Person- years of follow-up | 1, 057, 506 | 1,077, 203 | 2,414,412 | 2,575, 123 | 461,388 | 513,197 |
| Year of enrollment |  |  |  |  |  |  |
| Age at enrollment, mean, SD | 40 ± 8 | 40 ± 7 | 43 ± 6 | 43 ± 5 | 48 ± 15 | 48 ± 16 |
| Number LC deaths | 1181 | 686 | 1501 | 1288 | 641 | 405 |
| ≥13 years of education, (%) | 14 | 13 | 26 | 22 | 21 | 21 |
| Body mass index (BMI), mean, (kg/m2 ) | 25 | 24 | 26 | 24 | 26 | 25 |
| Level of physical activity, heavy (%) | 32 | 12 | 35 | 21 | 38 | 28 |
| Never smokers (%) | 26 | 46 | 34 | 39 | 38 | 44 |
| Former smokers (%) | 23 | 14 | 26 | 21 | 31 | 24 |
| Current smokers (%) | 51 | 40 | 40 | 40 | 31 | 32 |

^a^Enrollment period for each study
