# Supplementary material for: Smoking related lung cancer mortality by education and sex in Norway
Source: BMC Cancer. 2019 Nov 21;19:1132. doi: 10.1186/s12885-019-6330-9 (PMC6873553; doi:10.1186/s12885-019-6330-9)
Supplement: Supplementary file 2 — Additional file 2: Table S2. Hazard ratiosa (95% CIs) for lung cancer mortality according to categorical measures, for current smokers. [file 12885_2019_6330_MOESM2_ESM.docx]

Table S2. Hazard ratios^a^ (95% CIs) for lung cancer mortality according to categorical

measures, for current smokers

| Smoking status | Cases | Men  HR^a^ 95% CI | Cases | Women  HR^a^ 95% CI | Heterogeneity test for men versus women  P-values |
| --- | --- | --- | --- | --- | --- |
| Never | 91 | 1.00 (ref) | 188 | 1.00 (ref) |  |
| Duration of smoking, years | | | | | |
| 1-19 | 277 | 11.78 (9.26-14.98) | 333 | 7.29 (6.05-8.78) | <0.01 |
| 20-29 | 1626 | 20.01 (16.15-24.80) | 1300 | 15.18 (12.91-17.86) | 0.04 |
| ≥30 | 858 | 24.45 (19.50-30.66) | 336 | 21.71 (17.72-26.60) | 0.44 |
| p-trend |  | <0.001 |  | <0.001 |  |
| Cigarettes smoked per day | | | | | |
| 1-10 | 715 | 12.63 (10.14-15.73) | 820 | 10.04 (8.53-11.81) | 0.10 |
| 11-20 | 1536 | 23.90 (19.31-29.59) | 1035 | 20.45 (17.37-24.06) | 0.25 |
| ≥21 | 425 | 40.70 (32.38-51.16) | 119 | 37.30 (29.46-47.23) | 0.60 |
| p-trend |  | <0.001 |  | <0.001 |  |
| Pack-years | | | | | |
| 1-9 | 261 | 9.31 (7.32-11.84) | 363 | 6.50 (5.43-7.79) | 0.02 |
| 10-19 | 1027 | 17.61 (14.19-21.86) | 995 | 16.35 (13.91-19.22) | 0.59 |
| ≥20 | 1381 | 29.60 (23.90-36.67) | 607 | 26.64 (22.50-31.53) | 0.45 |
| p-trend |  | <0.001 |  | <0.001 |  |

^a^Adjusted for body mass index, physical activity level, all at enrollment, and duration of education
